# Supplementary material for: Climbing adaptations, locomotory disparity and ecological convergence in ancient stem ‘kangaroos’
Source: R Soc Open Sci. 2019 Feb 6;6(2):181617. doi: 10.1098/rsos.181617 (PMC6408368; doi:10.1098/rsos.181617)
Supplement: Supplementary material [file rsos181617supp1.docx]

Electronic supplementary material

**Climbing adaptations, locomotory disparity and ecological convergence in ancient stem ‘kangaroos’**

Wendy Den Boer^1,2^, Nicolás E. Campione^2,3,4^ and Benjamin P. Kear^5^

^1^Department of Palaeobiology, Swedish Natural History Museum, SE-104 05 Stockholm, Sweden.

^2^Department of Earth Science, Uppsala University, Villavägen 16, SE-752 36 Uppsala, Sweden.

^3^Department of Organismal Biology, Uppsala University, Norbyvägen 18A, SE-752 36 Uppsala, Sweden.

^4^Palaeoscience Research Centre, School of Environmental and Rural Sciences, University of New England, Armidale, New South Wales 2531, Australia.

^5^Museum of Evolution, Uppsala University, Norbyvägen 16, SE-752 36 Uppsala, Sweden.

**Author for correspondence:**

Benjamin P. Kear

email: benjamin.kear@em.uu.se

S1. Description of fossils

1.1. Fossil cataloguing

The specimens, QM F59022, QM F59023, QM F59024 and QM F59025 were initially recorded with the corresponding temporary QM cataloguing numbers QM F19717, QM F19718, QM F19719 and QM F19720, respectively (see figure 1), at the time of their preparation and storage at the University of New South Wales (Sydney, Australia) in 1998. However, these numbers were duplicates from Scanlon [1,2], and have been replaced herein with updated accession information formally issued by the QM (K. Spring [QM] pers. comm. 07/12/2018).

1.2. Astragali

QM F59022 is mediolaterally broad with a narrow, laterally sloping trochlear articular sulcus. The median trochlear crest is prominent and arches posteriorly onto the astragalus-calcaneum facet. Its lateral surface is rugose for attachment of the tibioastragalar ligament. The lateral trochlear crest is flush with the floor of the trochlear articular sulcus and does not continue onto the astragalus-calcaneum facet. The medial malleolar fossa (observable in both QM F59022 and QM F59023) is deep and oval in outline. Its lateral rim is greatly inflated and forms a continuous thickened border. The malleolar process is extremely pronounced and medially projecting. The astragalar neck in both QM F59022 and QM F59023 is elongate with a shallow dorsal astragalotibial ‘pit’ (= bursal/trochlear notch). The navicular facets are circular in outline when viewed anteriorly, but extend posteromedially as an elongate, ‘condyle-like’ articular surface (broken in QM F59023) that is obliquely deflected at 220º relative to a transverse plane through the trochlear articular surface. The plantar surface of QM F59022 is broad with an elongate medial calcanear facet. This is situated posteromedially relative to the transverse V-shaped trough for the ligamentum cervisis tali, which would have extended between the navicular facet and anterolateral plantar process in life.

1.3. Fibula

The incomplete distal fibula shaft of QM F59024 is elliptical in cross-section with a flattened medial surface for contact with the tibia. The distal epiphysis is rectangular in profile with an inset plantar midline groove for the peroneal tendon. A pair of prominent distolateral crests would have provided attachments for the superior peroneal retinaculum. The malleolar fossa is steeply inclined, and the adjacent anterior surface is rugose and inset for the flexor digitorum profundus and external lateral ligament.

1.4. Pedal digit IV ungual

The pedal digit IV ungual process of QM F59025 is laterally compressed and down-curved with a flattened plantar surface. The tapered tip is ‘squared-off’ distally, and the dorsal crest is weakly defined. The edges of the proximal articular surface are produced into a continuous raised flange. The plantar process is flattened and rectangular in outline with a shallow transverse groove for the flexor hallucus longus; this terminates in deeply inset lateral pits.

S2. Supplementary tables

**Table S1**. Measurements (mm) of individual balbarid-like macropodoid postcranial elements recovered from ‘Upper Site’ in the Riversleigh World Heritage Area of Queensland, Australia.

| **specimen** | **dimension** | **measurement** |
| --- | --- | --- |
| ***astragalus*** |  |  |
| QM F59022 | maximum length | 19 |
|  | maximum width | 21 |
|  | maximum length navicular facet | 9.6 |
| ***astragalus*** |  |  |
| QM F59023 | maximum length | 16 |
|  | maximum width | 12 |
| ***fibula*** |  |  |
| QM F59024 | maximum length | 30.9 |
|  | maximum anteroposterior diameter distal shaft | 9 |
|  | maximum anteroposterior length distal epiphysis | 10.5 |
| ***pedal digit IV ungual*** |  |  |
| QM F59025 | maximum length | 18 |
|  | maximum distal width | 4.5 |

**Table S2**. Taxon/specimen list and measurements (mm) of macropodoid astragali used for the analyses of 2D landmarks and linear metrics. ML, maximum anteroposterior length in dorsal view; MW, maximum mediolateral width in dorsal view; MD, maximum depth from the apex of the lateral trochlear crest; WTS, maximum mediolateral width across trochlear sulcus; LMTC, maximum length of the medial trochlear crest; LMTS, maximum length of the lateral trochlear crest; WNF, maximum width of the navicular facet; HNF, maximum height of the navicular facet. *Notamacropus rufogriseus* (AM S1635) was also included in the 2D landmarks dataset. Institutional abbreviations: AM, Australian Museum, Sydney; MV, Museums Victoria, Melbourne; QM, Queensland Museum, Brisbane.

| **category/taxon** | **specimen** |  | | **measurement** | | | | | | |
| --- | --- | --- | --- | --- | --- | --- | --- | --- | --- | --- |
|  |  | **ML** | **MW** | | **MD** | **WTS** | **LMTC** | **LLTC** | **WNF** | **HNF** |
| ***arboreal*** |  |  |  | |  |  |  |  |  |  |
| *Dendrolagus bennettianus* | AM S640 | 19 | 24 | | 9 | 12 | 17 | 12 | 9 | 6 |
| *Dendrolagus goodfellowi* | AM M9145 | 20 | 21 | | 12 | 15 | 20 | 17 | 7 | 6 |
| *Dendrolagus inustus* | AM M25390 | 17 | 18 | | 14 | 17 | 23 | 21 | 9 | 7 |
| *Dendrolagus lumholtzi* | AM M9108 | 16 | 24 | | 10 | 12 | 21 | 19 | 8 | 7 |
| *Dendrolagus matschiei* | MV C2917 | 15 | 21 | | 6 | 9 | 13 | 15 | 4 | 3 |
| *Dendrolagus dorianus* | AM M 9109 | 15 | 21 | | 10 | 13 | 18 | 17 | 10 | 6 |
| ***bipedal saltating*** |  |  |  | |  |  |  |  |  |  |
| *Aepyprymnus rufescens* | MV C17599 | 9 | 14 | | 7 | 7 | 9 | 11 | 6 | 4 |
| *Bettongia gaimardi* | MV C28683 | 11 | 13 | | 7 | 7 | 10 | 12 | 4 | 3 |
| *Potorous tridactylus* | MV C26002 | 4 | 7 | | 3 | 5 | 7 | 9 | 3 | 2 |
| *Dorcopsis luctuosa* | MV C31253 | 17 | 17 | | 12 | 10 | 14 | 15 | 5 | 6 |
| *Dorcopsis muelleri* | MV C28830 | 15 | 20 | | 9 | 11 | 19 | 17 | 4 | 8 |
| *Lagostrophus fasciatus* | MV C6792 | 7 | 12 | | 6 | 6 | 10 | 9 | 3 | 4 |
| *Onychogalea fraenata* | AM M38911 | 13 | 16 | | 7 | 10 | 14 | 13 | 3 | 5 |
| *Onychogalea unguifera* | MV C6407 | 14 | 17 | | 12 | 10 | 14 | 11 | 4 | 7 |
| *Osphranter robustus* | MV C23544 | 33 | 30 | | 20 | 12 | 44 | 30 | 7 | 14 |
| *Osphranter rufus* | MV R4280 | 39 | 37 | | 26 | 19 | 50 | 35 | 7 | 14 |
| *Petrogale brachyotis* | MV C11593 | 14 | 21 | | 7 | 8 | 19 | 13 | 5 | 4 |
| *Petrogale godmani* | MV C26530 | 16 | 20 | | 11 | 10 | 18 | 14 | 4 | 6 |
| *Petrogale penicillata* | MV C6425 | 19 | 21 | | 12 | 11 | 20 | 13 | 6 | 7 |
| *Petrogale xanthopus* | MV C9486 | 21 | 20 | | 9 | 12 | 22 | 13 | 7 | 8 |
| *Macropus fuliginosus* | MV C9504 | 34 | 38 | | 21 | 21 | 43 | 32 | 9 | 14 |
| *Macropus giganteus* | MV C6465 | 36 | 39 | | 25 | 24 | 48 | 32 | 7 | 16 |
| *Notamacropus eugenii* | MV C17615 | 14 | 13 | | 7 | 8 | 15 | 13 | 4 | 6 |
| *Notamacropus parryi* | AM M23587 | 26 | 30 | | 14 | 19 | 24 | 16 | 9 | 10 |
| *Setonix brachyurus* | MV C6446 | 11 | 14 | | 4 | 7 | 10 | 11 | 3 | 2 |
| *Thylogale billardierii* | MV C7995 | 17 | 19 | | 10 | 5 | 16 | 20 | 7 | 7 |
| *Wallabia bicolor* | MV C10742 | 22 | 26 | | 15 | 15 | 26 | 23 | 7 | 11 |
| ***obligate quadrupedal*** |  |  |  | |  |  |  |  |  |  |
| *Hypsiprymnodon moschatus* | AM M40093 | 7 | 9 | | 6 | 4 | 7 | 7 | 3 | 4 |
| ***fossil*** |  |  |  | |  |  |  |  |  |  |
| astragalus | QM F59022 | 19 | 21 | | 10 | 14 | 17 | 16 | 5 | 6 |
| *Nambaroo gillespieae* | QM F34532 | 16 | 17 | | 9 | 8 | 11 | 11 | 6 | 8 |

**Table S3**. Taxon/specimen list of macropodoid pedal digit IV unguals used for the analyses of 2D landmarks.

| **category/taxon** | **specimen** |
| --- | --- |
|  |  |
| ***arboreal*** |  |
| *Dendrolagus bennettianus* | AM S640 |
| *Dendrolagus goodfellowi* | AM M9145 |
| *Dendrolagus inustus* | AM M25390 |
| *Dendrolagus lumholtzi* | AM M9108 |
| *Dendrolagus matschiei* | AM M9146 |
| *Dendrolagus dorianus* | AM M9109 |
| *Dendrolagus dorianus* | AM M7406 |
| ***bipedal saltating*** |  |
| *Aepyprymnus rufescens* | AM S51308 |
| *Bettongia gaimardi* | MV C28683 |
| *Bettongia penicillata* | AM M36576 |
| *Bettongia tropica* | AM M40087 |
| *Potorous longipes* | AM M41268 |
| *Dorcopsis muelleri* | AM M32341 |
| *Lagorchestes hirsutus* | AM M38909 |
| *Onychogalea fraenata* | AM M38911 |
| *Osphranter rufus* | AM M33372 |
| *Petrogale lateralis* | AM M38619 |
| *Petrogale penicillata* | AM S1217 |
| *Petrogale xanthopus* | AM M38625 |
| *Macropus fuliginosus* | AM M3337 |
| *Macropus giganteus* | AM M37235 |
| *Notamacropus agilis* | AM M9164 |
| *Notamacropus dorsalis* | AM M51193 |
| *Notamacropus eugenii* | AM M39928 |
| *Notamacropus parma* | AM M33808 |
| *Notamacropus parryi* | AM M23587 |
| *Notamacropus rufogriseus* | AM S1635 |
| *Setonix brachyurus* | AM M18626 |
| *Thylogale stigmatica* | AM M38878 |
| *Thylogale thetis* | AM M1042 |
| *Wallabia bicolor* | AM M32986 |
| ***obligate quadrupedal*** |  |
| *Hypsiprymnodon moschatus* | MV C6785 |
| ***fossil*** |  |
| pedal digit IV ungual | QM F59025 |
| *Nambaroo gillespieae* | QM F34532 |

**Table S4**. Standard deviations (SD), proportions of variance and cumulative percentages for each Principal Component (PC) derived from analyses of the astragalar 2D landmark datasets.

| **value** | **PC1** | **PC2** | **PC3** | **PC4** | **PC5** | **PC6** | **PC7** | **PC8** | **PC9** | **PC10** |
| --- | --- | --- | --- | --- | --- | --- | --- | --- | --- | --- |
| ***trochlear*** |  |  |  |  |  |  |  |  |  |  |
| SD | 0.06 | 0.05 | 0.04 | 0.03 | 0.02 | 0.02 | 0.02 | 0.02 | 0.02 | 0.01 |
| variance | 0.32 | 0.25 | 0.13 | 0.08 | 0.05 | 0.04 | 0.03 | 0.03 | 0.02 | 0.01 |
| cumulative | 0.32 | 0.56 | 0.69 | 0.77 | 0.82 | 0.86 | 0.89 | 0.92 | 0.94 | 0.95 |
| ***medial*** |  |  |  |  |  |  |  |  |  |  |
| SD | 0.1 | 0.07 | 0.06 | 0.05 | 0.04 | 0.03 | 0.03 | 0.02 | 0.02 | 0.01 |
| variance | 0.39 | 0.2 | 0.12 | 0.1 | 0.05 | 0.04 | 0.04 | 0.02 | 0.02 | 0.01 |
| cumulative | 0.39 | 0.59 | 0.7 | 0.8 | 0.85 | 0.89 | 0.93 | 0.95 | 0.96 | 0.97 |
| ***anterior*** |  |  |  |  |  |  |  |  |  |  |
| SD | 0.07 | 0.06 | 0.05 | 0.04 | 0.03 | 0.03 | 0.02 | 0.02 | 0.02 | 0.01 |
| variance | 0.31 | 0.2 | 0.17 | 0.1 | 0.05 | 0.04 | 0.03 | 0.02 | 0.02 | 0.01 |
| cumulative | 0.31 | 0.51 | 0.68 | 0.78 | 0.83 | 0.87 | 0.9 | 0.92 | 0.93 | 0.94 |
| ***plantar*** |  |  |  |  |  |  |  |  |  |  |
| SD | 0.06 | 0.06 | 0.05 | 0.03 | 0.03 | 0.02 | 0.02 | 0.01 | 0.01 | 0.01 |
| variance | 0.31 | 0.26 | 0.18 | 0.07 | 0.05 | 0.04 | 0.02 | 0.02 | 0.01 | 0.01 |
| cumulative | 0.31 | 0.57 | 0.75 | 0.82 | 0.87 | 0.9 | 0.93 | 0.94 | 0.96 | 0.97 |
| ***lateral*** |  |  |  |  |  |  |  |  |  |  |
| SD | 0.08 | 0.08 | 0.07 | 0.04 | 0.03 | 0.03 | 0.02 | 0.02 | 0.02 | 0.02 |
| variance | 0.3 | 0.25 | 0.19 | 0.06 | 0.04 | 0.03 | 0.02 | 0.02 | 0.02 | 0.01 |
| cumulative | 0.3 | 0.55 | 0.74 | 0.8 | 0.84 | 0.88 | 0.9 | 0.92 | 0.94 | 0.96 |
| ***posterior*** |  |  |  |  |  |  |  |  |  |  |
| SD | 0.1 | 0.08 | 0.05 | 0.04 | 0.03 | 0.02 | 0.02 | 0.02 | 0.02 | 0.02 |
| variance | 0.43 | 0.25 | 0.1 | 0.06 | 0.04 | 0.02 | 0.02 | 0.01 | 0.01 | 0.01 |
| cumulative | 0.43 | 0.69 | 0.79 | 0.86 | 0.9 | 0.92 | 0.94 | 0.95 | 0.96 | 0.97 |

**Table S5**. Standard deviations, proportions of variance and cumulative percentages for each PC derived from analyses of the astragalar linear measurement dataset.

| **value (%)** | **PC1** | **PC2** | **PC3** | **PC4** | **PC5** | **PC6** | **PC7** | **PC8** |
| --- | --- | --- | --- | --- | --- | --- | --- | --- |
| SD | 0.52 | 0.12 | 0.11 | 0.09 | 0.08 | 0.06 | 0.04 | 0.04 |
| variance | 0.85 | 0.05 | 0.04 | 0.02 | 0.02 | 0.01 | 0.01 | 0.01 |
| cumulative | 0.85 | 0.9 | 0.9 | 0.96 | 0.98 | 0.99 | 0.99 | 1 |

**Table S6**. Standard deviations, proportions of variance and cumulative percentages for each PC derived from analyses of the pedal digit IV ungual 2D landmark datasets.

| **value (%)** | **PC1** | **PC2** | **PC3** | **PC4** | **PC5** | **PC6** | **PC7** | **PC8** | **PC9** | **PC10** |
| --- | --- | --- | --- | --- | --- | --- | --- | --- | --- | --- |
| ***dorsal*** |  |  |  |  |  |  |  |  |  |  |
| SD | 0.09 | 0.04 | 0.03 | 0.02 | 0.01 | 0.01 | 0.01 | 0.0 | 0.0 | 0.0 |
| variance | 0.74 | 0.12 | 0.07 | 0.03 | 0.02 | 0.01 | 0.0 | 0.0 | 0.0 | 0.0 |
| cumulative | 0.74 | 0.85 | 0.93 | 0.96 | 0.98 | 0.99 | 0.99 | 1 | 1 | 1 |
| ***lateral*** |  |  |  |  |  |  |  |  |  |  |
| SD | 0.07 | 0.06 | 0.04 | 0.03 | 0.02 | 0.01 | 0.01 | 0.01 | 0.01 | 0.0 |
| variance | 0.44 | 0.31 | 0.13 | 0.06 | 0.02 | 0.01 | 0.01 | 0.01 | 0.0 | 0.0 |
| cumulative | 0.44 | 0.76 | 0.89 | 0.95 | 0.97 | 0.98 | 0.99 | 0.99 | 1 | 1 |

S3. Supplementary figure

**

**

**Figure S1**. Landmark placement and measurements. (*a*) Astragalar semi-, (⚫) and fixed (🞆) landmarks dataset. (*b*) Pedal digit IV ungual semi (⚫), and fixed (🞆) landmarks dataset. (*c*) Astragalar linear measurements dataset in trochlear (top), and anterior view (bottom). Astralagar views in (*a*) are trochlear, medial, and anterior (left top to bottom); and plantar, lateral, and posterior (right top to bottom). Pedal digit IV ungual process is depicted in dorsal (left) and medial (right) views in (*b*). Abbreviations for measurement parameters in (*c*) are listed in table S2. Graphics produced with *Adobe* CC2016 by W.D.B. and B.P.K.

S4. Supplementary references

1. Scanlon JD. 1993 Madtsoiid snakes from the Eocene Tingamarra Fauna of eastern Queensland. *Kaupia* **3**, 3–8.
2. Scanlon JD. 2005 Australia’s oldest known snakes: *Patagoniophis*, *Alamitophis*, and cf. *Madtsoia* (Squamata: Madtsoiidae) from the Eocene of Queensland. *Mem. Queensl. Mus.* **51**, 215–235.
